# Medical students’ adoption and evaluation of a completely digital general practice clerkship – cross-sectional survey and cohort comparison with face-to-face teaching

**DOI:** 10.1080/10872981.2022.2028334

**Published:** 2022-02-02

**Authors:** Marina Fehl, Vera Gehres, Anne-Kathrin Geier, Thomas Mundt, Kay Klinge, Thomas Frese, Markus Bleckwenn, Tobias Deutsch

**Affiliations:** a Department of General Practice, Faculty of Medicine, University of Leipzig, Leipzig, Germany; b Institute of General Practice and Family Medicine, Martin-Luther-University Halle-Wittenberg, Halle/Saale, Germany

**Keywords:** Undergraduate medical education, general practice, clerkship, online teaching, blended learning

## Abstract

**Background:**

During the COVID-19 pandemic, the University of Leipzig completely switched to online teaching. Thus, we developed a practice-oriented digital substitute for a two-week mandatory general practice (GP) clerkship. Main components were processing of clinical cases and additional GP topics, visual diagnoses, information and examination videos, and regular remote exchanges with associated GP teachers. We took the chance to comprehensively evaluate the new teaching formats (acceptance, use, working enjoyment, learning gain, practical relevance, insights into general practice) and to compare evaluations with two previous semesters to gain insights for future blended learning concepts.

**Methods:**

Cross-sectional post-hoc online evaluation among fourth year (of six) medical students participating in the digital mandatory 2-week GP clerkship during summer semester 2020; additional cohort comparison with two previous semesters (face-to-face clerkship).

**Results:**

Out of 192 participants in the digital clerkship, 99 completed our questionnaire (response rate = 51.6%). Results were compared with 277 previous evaluations (face-to-face semesters). Most participants reported having enjoyed the online-based clerkship (87.9%), having learned a lot (89.9%), having gained insights into general practice (76.8%), and perceived high practical relevance (90.9%). Implementing the new teaching formats into future face-to-face clerkships was welcomed by 65.6%. Clinical cases, visual diagnoses, examination videos and communication with GP teachers were rated best regarding working enjoyment, learning gain, practical relevance and insights into a GP’s work. Cohort comparison revealed somewhat better evaluations regarding knowledge transfer for the digital clerkship while imparting of skills and attitudes was reportedly worse.

**Conclusions:**

Students welcomed the digital content and perceived relevant learning gain. Our results may help to develop future blended learning concepts. Clinical cases, examination videos and visual diagnoses appear to be particularly suitable as useful online complements which could enrich face-to-face teaching. As students especially valued the exclusive time for exchanges with their preceptor, this should be facilitated in face-to-face clerkships.

## Introduction

During the COVID-19 pandemic, universities around the world were forced to switch to online teaching entirely or partially [1,2]. Unlike other German medical faculties, medical students at the University of Leipzig were not even permitted to complete their mandatory 2-week general practice (GP) clerkship as usual. To enable the students to follow a normal course of study, we developed a practice-oriented digital substitute for the clerkship within 4 weeks. Apart from all restrictions and contradictions of an online-based ‘practice experience’ we took the chance to test different online teaching formats and to evaluate the potential to complement GP clerkships also after the pandemic. In Germany, undergraduate medical education in general practice is expected to be substantially expanded in the coming years to enlarge the representation of primary health care during medical studies and to attract more students to GP careers (‘Masterplan 2020’, upcoming amendment of the licensing regulations for doctors, presumably beginning in 2025) [3]. In addition to longitudinal GP teaching during the whole course of studies, the amount and extent of GP clerkships will increase. Consequently, the development of innovative teaching concepts is of particular importance. The planned extension of the GP clerkship duration may offer the opportunity to integrate digital learning content, while still having enough time for face-to-face teaching (blended learning).

Undergraduate medical education in Germany has a duration of 6 years. It is currently divided into a 2-year pre-clinical (more theoretical) study section, a 3-year clinical (more practice-oriented) study section and the final practical year [4]. In recent decades, undergraduate education in general practice has increasingly been established at German medical faculties [5]. Today, general practice is a mandatory subject for all students in the clinical study section. In addition to a lecture series ending with an examination, all students have to complete a 2-week GP clerkship (‘Blockpraktikum’) in an associated academic GP teaching practice and a 4-week clerkship in ambulatory primary care (‘Famulatur’) in any practice providing general practice, general internal medicine, or pediatrics [6,7]. Practical teaching in general practice is organized in a decentralized manner in collaboration with networks of so-called academic teaching practices. The respective GPs are officially associated with the university and provide 1:1 supervision of students in their practices.

To develop future concepts for practice-oriented blended learning in medical education and particularly in general practice, more evidence is needed regarding the potential of different online teaching formats [8]. This study investigated how medical students participating in a completely online-based GP clerkship accepted, used, and evaluated the new format and its individual components in terms of working enjoyment, learning gain, practical relevance and insights into general practice. To reveal advantages and disadvantages of the online-based compared to the conventional face-to-face clerkship, it was of further interest how these evaluations differed from those of two previous semesters.

## Materials and methods

### Sampling and design

We conducted a cross-sectional survey among 4^th^ year medical students at the University of Leipzig who completed their online-based mandatory GP clerkship between April and June 2020. Analogous to the face-to-face clerkship, there was a processing period of 2 weeks with a total processing time of approximately 30 hours. Five processing periods were offered, and each of them was attended by about 40 students. Altogether, 192 students took part in the online-based clerkship.

Prior to working through the online content, each student was assigned to an individual GP teacher (decentral associated academic teaching practice).

Immediately after finishing the clerkship, students were invited via e-mail to participate in an anonymous and voluntary online evaluation.

To enable a comparison between students who completed the digital clerkship and those who took part in the conventional one, all available anonymized evaluations (identical items) of the two previous semesters were added to the data set (n = 277 paper-based evaluations between April 2019 and March 2020, response rate = 88.2%).

### Content and structure of the digital clerkship

**Figure 1.**
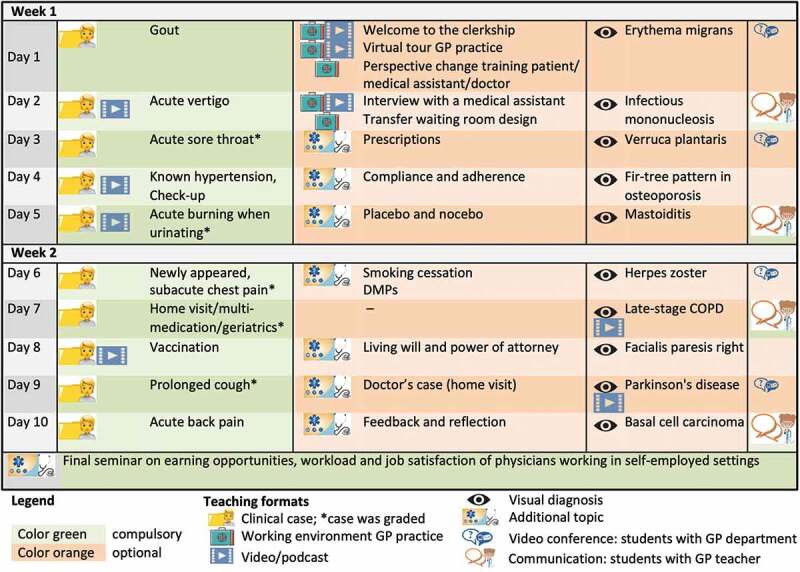
Chronological overview of the teaching content and formats during the 2-week online-based GP clerkship.

The aim of the online-based clerkship was to provide students an insight into general practice with its particularities regarding patient clientele, spatial conditions and economic and organizational structure despite the lack of physical presence. The core of our digital clerkship consisted of 10 clinical SOAP*-cases partly linked with videos, based on which the students would have a professional exchange with their individual GP teacher. In addition, there were numerous materials with which the students would learn about general practice. Figure 1 gives a detailed sequential overview of all components and teaching formats included. The processing time (time needed for task completion) for the individual components varied from about 5 minutes for the visual diagnoses (recognition of a health disorder or pathological change by simply looking at it) to up to 4 hours for individual clinical cases. The total processing time was about 30 hours. In order to gain at least some practical experience, students were encouraged to conduct physical examinations of their flatmates or family members, as well as simulated doctor patient consultations to take medical histories or conduct therapy interviews by telephone with relatives and acquaintances. As in the conventional clerkship, every student was supervised and graded individually by a GP teacher working in an associated teaching practice. Grading was based on processing of 5 out of 10 clinical cases and the GP teacher’s personal impression of the student’s understanding of general practice in the remote conversations. For reasons of verification and archiving, the students were required to send the processed documents using a personalized upload link. All communication during the clerkship was completely digital. Organizational information for students and GP teachers was provided via e-mail and the learning material for the students was available via the university’s official student portal. Despite known better functionalities of other learning platforms, we decided to use the student portal because the students were familiar with its operation. The GP teachers could access all teaching material via cloud and received ideal sample solutions for the clinical cases by e-mail. Communication between students and GP teachers was possible via video chat, telephone, and e-mail. Overall, we wanted to ensure the greatest possible usability to support good acceptance of the new format by students and GP teachers and to avoid possible dysfunctionalities. According to the findings of Chu et al., maximal usability and simplicity are among the most important factors to support the adoption of e-learning systems [9].

We aimed to achieve the greatest possible depth of learning and variety for the students by using a wide range of different teaching methods. To maintain continuous contact with students and to provide them with support for the new format, we offered four video live chats (about 30 min duration). In one of them, the students had the opportunity to talk to the head of our GP department. Except for these live chats, the completion of the clerkship was flexible in terms of time allowing an asynchronous processing of all tasks. However, sequential processing was recommended.

At the end of our conventional GP clerkship, students usually attended a seminar dealing with earning opportunities, workload and job satisfaction of physicians working in self-employed settings. This was also implemented online in the form of a screencast.

*) The Subjective, Objective, Assessment and Plan (SOAP) note is an acronym representing a widely used method of documentation for healthcare providers [10,11].

### Questionnaire

A multidisciplinary team including physicians and social scientists developed the two-part questionnaire. The first part focused on the online-based clerkship and contained questions addressing the following topics: socio-demographics, career considerations, overall assessments regarding the online-based clerkship, assessments of the individual components regarding working enjoyment, learning gain, practical relevance and insight into a GP’s work, and user behavior. To enable comparability with the evaluation of the conventional clerkship in previous semesters, the second part contained items identical to the evaluation used in previous years related to the clerkship in general, assessments of the teaching of new skills and attitudes, and the GP teacher-student relationship. In addition, the students had the opportunity to state what they liked about the clerkship and what should be improved in form of free text. The final questionnaire was completed online and took about 10 minutes. An English translation of the questionnaire items analyzed in this study is presented in Supplemental File 1.

### Statistical analysis

Data were analyzed using IBM SPSS Statistics 24 for Windows. Continuous variables were presented as mean ± SD while frequencies were presented as %_valid_ (n_absolute_/n_valid_) considering missing values for individual items. Besides descriptive analysis, one-sample χ^2^ test was used to compare frequency distributions between independent groups. Due to the absence of normal distribution, Mann-Whitney U test was used to compare differences in central tendency. Statistical significance was assumed at p <0.05.

### Qualitative analysis of the free text answers

At the end of the questionnaire the students had the opportunity to state in free text what they liked about the online-based clerkship and what should be improved. The resulting qualitative data were analyzed according to Mayring’s qualitative content analysis [12]. In a first step, two scientists (GP residents) developed categories independently from each other following an inductive approach and including all available material. The resulting category systems were compared, and consensus was found for all differences. Applicable categories were used only once per person. To be able to assess the reliability of the results, a third previously uninvolved rater (GP resident) allocated the raw data once again to the category systems. Interrater agreement was 89.7% for the first question (what students liked) and 85.0% for the second question (what should be improved), which can be considered as reliable due to a high number of categories (first question 14, second question 17). Finally, frequencies of mentioning were counted for each category.

### Ethics approval

According to the regulations of the local ethics committee (Ethical Committee at the Faculty of Medicine of the University of Leipzig) and in reference to the Model Professional Code of Conduct for physicians working in Saxony, Germany [13], an explicit ethical approval was deemed unnecessary for this study based on anonymous questionnaires without allowing the identification of individuals. All participants were informed about the background and intention of the study and participation was completely voluntary.

## Results

### Study participation and sample characteristics

Overall, 99 out of 192 students participating in the online-based clerkship completed the questionnaire (response rate = 51.6%). The socio-demographic sample characteristics are shown in Table 1.

**Table 1.** Sample characteristics

**Variable**		Valid (n)	n (%)^1)^
Female gender		98	68 (69.4)
Age (mean ± SD)		98	24.8 ± 3.7
Study year		99	
	… 4th		88 (88.9)
	… 5th (catch-ups)		11 (11.1)
Pre-existing completed medical vocational training		97	20 (20.6)
Has already completed 4-week clerkship in general practice		99	69 (69.7)
Being a physician’s child		99	23 (23.2)
Family or friends working as GP		99	32 (32.3)
Mainly grew up in …		98	
	… big city		29 (29.6)
	… small town		38 (38.8)
	… rural area		31 (31.6)

Note: ^1)^Unless otherwise indicated.

Altogether, 87.9% (87/99) rated themselves as *‘*very fit’ or ‘rather fit’ in using computers, 12.1% (12/99) felt ‘rather unfit’, with none describing themselves as ‘not fit at all’. Although not statistically significant due to the small sample size, 11 of the 12 persons describing themselves as ‘rather unfit’ were women.

For 7.1% (7/99) general practice was the currently favored career and for 73.7% (73/99) it was at least a considerable option. Being self-employed in one’s private practice was the favored option for 25.3% (25/99) and basically imaginable for 64.6% (64/99) of the participants. While 37.4% (37/99) preferred to work in ambulatory health care in the future it was a considerable option for 61.6% (61/99) and 1.0% (1/99) could not imagine ambulatory care at all.

### Overall evaluation of the online-based GP clerkship

**Figure 2.**
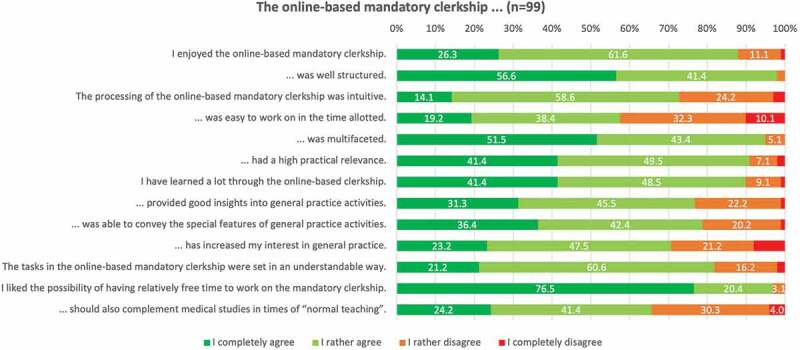
Medical students’ overall assessment of the whole online-based GP clerkship.

Figure 2 gives an overview of how the students evaluated the online-based clerkship in general. The possibility for flexible time management, the structure, and the multifaceted nature of the learning content were rated particularly positively, followed by perceived practical relevance, learning gain, and working enjoyment (‘completely agree’ and ‘rather agree’).

### Usage behavior

Most students used a laptop for completing their work (89.9% (89/99)). Tablet computers (22.2% (22/99)), smartphones (19.2% (19/99)) and desktop PCs (12.1% (12/99)) were used far less frequently (multiple answers were possible). Altogether, 70.7% (70/99) reported having worked on the clerkship daily and 93.9% (93/99) completed the components sequentially in the intended chronological order (‘yes’ or ‘rather yes’). Joint work with fellow students was reported by 41.4% (41/99), and 28.3% (28/99) stated having practiced examinations on others.

**Figure 3.**
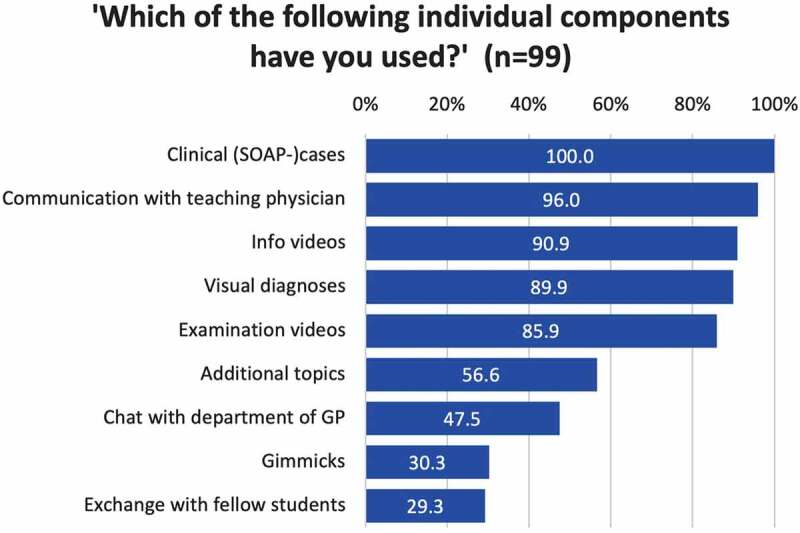
Medical students’ usage of the individual components (teaching formats) of the online-based GP clerkship in descending order by frequency of use.

Figure 3 shows the usage behavior regarding the individual components (teaching formats) of the online-based clerkship. The mandatory clinical cases and exchanges with the GP teacher were used by all, and nearly all, participants, respectively. Among the optional formats, students most frequently reported having seen the information and examination videos and having dealt with the visual diagnoses.

### Evaluation of individual teaching formats

**Figure 4.**
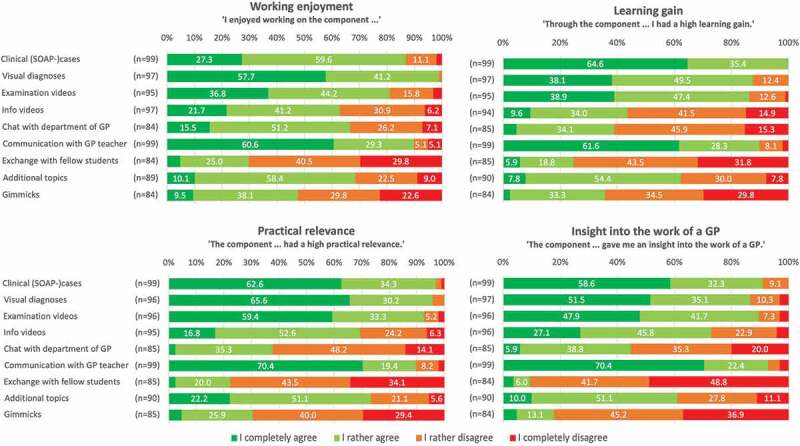
Medical students’ assessment of the online-based GP clerkship’s individual teaching formats regarding working enjoyment, learning gain, practical relevance and insights into a GP’s work.

Students’ assessments of each of the teaching formats in terms of working enjoyment, learning gain, practical relevance and insights into a GP’s work are displayed in Figure 4. All four aspects were rated best for clinical cases, visual diagnoses, examination videos and for the communication between student and GP teacher, followed by additional topics (e.g., smoking cessation, living will) and chat with department staff. To get insights into a GP’s work, students also rated the information videos (e.g., interview with a medical assistant) as useful. Exchange with fellow students and gimmicks (e.g., different types of puzzles) were rated as less helpful by the participants.

### Cohort comparison online-based vs. conventional clerkship

Evaluations of the students participating in the online-based clerkship (n = 99) were compared with those of two previous semesters (conventional face-to-face clerkship, n = 277). The two cohorts were structurally comparable regarding age (mean ± SD: 24.8 ± 3.7 vs. 24.8 ± 3.4 years, p = 0.615), proportion of women (69.4% vs. 59.7%, p = 0.090), and being a physician’s child (23.2% vs. 28.4%, p = 0.321).

The results of the cohort comparison of students’ assessments are presented in Table 2. There were no significant differences regarding students’ overall satisfaction, delivery on expectations and perception of the effort-benefit ratio. While the online-based clerkship was perceived as significantly more theoretical, professional exchange and learning were rated better. Regarding the acquisition of new skills and attitudes, students’ ratings for most items were superior in the conventional clerkship.

**Table 2.** Comparison of medical students’ assessments of the online-based (n = 99) versus the conventional (n = 277) 2-week GP clerkship

	Online-based	Conventional	Significance	Online-based vs. conventional*
Variable	mean ± SD	mean ± SD	p	
**General**	scale: 1 = totally … 6 = not at all			
My expectations regarding the goals and topics of the mandatory clerkship have been fulfilled.	2.3 ± 1.0	2.3 ± 1.3	0.261	=
During the mandatory clerkship I learned professionally.	1.8 ± 0.9	2.5 ± 1.3	< 0.001	+
During the mandatory clerkship, there was the opportunity for a professional exchange with my teaching physician.	1.4 ± 0.9	1.9 ± 1.3	< 0.001	+
The mandatory clerkship encouraged me to further deepen my self-study of the topics covered.	2.4 ± 1.4	2.9 ± 1.4	0.001	+
Measured in terms of time and organizational effort, participation in the mandatory clerkship was worthwhile.	2.4 ± 1.3	2.6 ± 1.5	0.191	=
**How well or poorly were you taught new skillsor attitudes in your mandatory clerkship on the following topics?**	scale: 1 = very well … 6 = very poorly			
Detection of common diseases in general practice and their therapy	1.8 ± 0.8	2.0 ± 1.1	0.047	+
Prescriptions (prescriptions, medicines, physiotherapy, incapacity to work, etc.)	3.5 ± 1.3	2.6 ± 1.3	< 0.001	-
Preventive measures (prevention of a later serious illness, e.g., hypercholesterolemia, obesity)	2.2 ± 1.0	2.5 ± 1.3	0.135	=
Screening measures (preventive examinations, e.g., check-ups, cancer screening)	2.6 ± 1.1	2.2 ± 1.3	0.001	-
Importance of family medicine	2.9 ± 1.3	2.7 ± 1.4	0.093	=
Home visits (indication, procedure, frequency)	2.5 ± 1.2	2.6 ± 1.5	0.643	=
Communication/conversation skills (also with difficult patients, compliance problems)	3.3 ± 1.5	2.2 ± 1.5	< 0.001	-
Meeting patient expectations	3.2 ± 1.3	2.2 ± 1.2	< 0.001	-
Care of the chronically ill	2.6 ± 1.2	2.0 ± 1.1	< 0.001	-
Vaccinations	2.3 ± 1.1	1.8 ± 1.3	< 0.001	-
Physical examination techniques	3.2 ± 1.3	2.3 ± 1.4	< 0.001	-
Instrumental diagnostics in general practice	3.4 ± 1.3	2.8 ± 1.5	< 0.001	-
**Questions about contact with the supervising teaching physician**	scale: 1 = totally … 10 = not at all			
I was motivated by the teacher to become a general practitioner myself later.	4.1 ± 2.6	5.0 ± 2.9	0.016	+
The teaching physician was able to explain everything well to me.	2.0 ± 2.0	2.7 ± 2.3	< 0.001	+
The clerkship was far too theoretical.	5.7 ± 2.6	8.5 ± 1.9	< 0.001	+
The demands on me were too high.	7.0 ± 2.3	8.7 ± 2.0	< 0.001	+
Overall, I am satisfied with the quality of the mandatory clerkship.	2.8 ± 1.9	3.4 ± 2.7	0.474	=

*+ higher rating in online-based compared to conventional, = same rating …, – lower rating …

### Qualitative analysis of the free text answers

**Figure 5.**
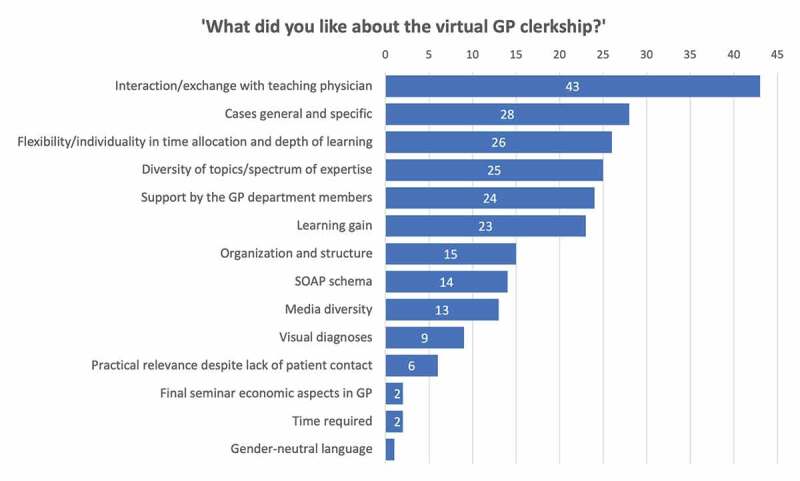
Qualitative analysis of students’ free text answers. The inductively generated categories were sorted in descending order by frequency of students’ mentioning of them.

Figure 5 shows the results of the qualitative analysis of the participants’ free text answers on what they liked about the clerkship and what could be improved. Categories were sorted in descending order by frequency of students’ mentioning. Participants reinforced their appreciation of the exchange with the GP teachers and the clinical cases. Students’ recommendations on what could be improved mostly referred to a reduction of processing time and scope of the clerkship and to restructure and optimize the clinical cases regarding content.

## Discussion

### Summary of the main findings

Our online-based GP clerkship was well accepted by the students. Overall, evaluations were particularly positive regarding flexible time management, structure, multifaceted nature of the learning content, perceived practical relevance, learning gain, and working enjoyment. Evaluations of the individual teaching components revealed that particularly explicit time slots for exchange with the GP teacher as well as online clinical cases, visual diagnoses, examination videos and dealing with additional topics typical for general practice (e.g., living will, smoking cessation) are ofhigh value from the students’ perspective. Compared with the face-to-face clerkship, the online-based clerkship was assessed similarly well overall. While there was a better evaluation for the acquisition of theoretical content, the training of practical content was mostly rated worse for the online-based version.

### Main findings in relation to other studies

#### Sample and computer literacy

Overall, the characteristic distributions of the sample in our study indicate representativeness and correspond to the nationwide data in terms of age and gender [14].

Students’ technical skills are important in assessing whether online teaching is feasible and accepted. It has been shown that online teaching is poorly accepted if students have little previous experience [15]. The fact that in our study almost all students felt confident shows that German students are well prepared for online learning.

#### General acceptance and usage behavior

Many principles of ‘good online teaching’ were met by our concept: clear learning objectives matching the curriculum, opportunities for synchronous and asynchronous teacher-student interaction, promotion of higher-order thinking skills and communication skills, encouragement of active and self-directed learning while promoting timely completion of tasks and effective time management [16]. This may have contributed to students’ overall satisfaction. Our results indicate thatwe have succeeded in providing practice relevant, well-structured, enjoyable, and multifaceted teaching content. The results also show that even without the presence of the students in the GP practice, it was possible to give the majority of students an insight into a GP’s work and to increase their interest in the specialty. Very well rated issues were the flexibility and individuality in time allocation and depth of learning during the online-based clerkship – an advantage of online teaching that has been described in many studies [2,17–20].

Our results imply that a substantial number of students had difficulties completing the tasks in the time allotted. It has been previously demonstrated that faculty tend to underestimate the workload of online assignments and that unclear information may be obstructive to a good online teaching experience [19]. Thus, it seems advisable to pre-test ease of use and determine the amount of processing time needed with students before implementing complex online teaching concepts.

The fact that two thirds of students support the implementation of our digital contents in future face-to-face clerkships shows the potential for blended learning beyond the current pandemic. A recent review of online teaching underlined that supplemented online content can provide additional benefit in undergraduate medical education [21].

In line with other current studies [22,23], most of the students in our sample preferred using their laptops to work on the assignments. This could have been partially due to the file formats used, which made processing via laptop (bigger screen, keyboard) more comfortable. As shown in previous studies, most medical students own mobile devices (smartphone/tablet), which could also be used for appropriate learning material [24].

Although not significant due to the small number of cases, it should be noted that 11 out of 12 participants in our study feeling ‘rather unfit’ in dealing with computers were female. According to Zilian and Zilian, female gender correlates negatively with digital problem-solving skills, although this effect is no longer evident in everyday use of information and communication technologies (ICT), which we deemed our digital format to be [25]. Nevertheless, our results indicate that some (female) students might need special technological support to successfully take part in online learning.

Although a non-chronological order would have been possible, most students worked sequentially and daily on the materials provided in our clerkship and the vast majority did so in chronological order. On the one hand, our default of daily tasks may have implied sequential processing. It has been shown that students find clear time structures in online teaching helpful [2]. On the other hand, sequential working might be the approach students are familiar with.

#### Usage and evaluation of the individual components

The provided clinical patient cases were relevant for examination and consequently used by all students. In parallel, the case work was among the best rated content in terms of working enjoyment, learning gain, practical relevance, and insight into GP activities. This corroborates results of former studies indicating that solving patient cases, e.g., in the course of problem-based learning sessions, is a highly welcomed teaching method [26–28].

Discussing cases with the GP teacher was the basis of grading and thus the second compulsory element. Nevertheless, few students stated difficulties in establishing contact with their GP teacher. However, for the overwhelming majority, the exchange with the GP preceptors worked surprisingly well for the first-time implementation of an online GP clerkship. The exchange with the GP teachers was rated particularly positively in all four evaluation categories. Also, students’ free text answers on what they liked the most revealed the particular importance of the student-GP interaction. This is in line with findings from both education and medical didactics. Video conferencing and student-teacher communication have been described to be perceived as highly beneficial [19,29]. It should be noted that involving GP preceptors in online teaching requires significant effort. It was not certain in advance whether the GPs would be willing to participate and whether online contact with the students could be integrated into their daily work routine. Anyway, GPs in our study were willing to try out the new format and our results show that this effort is worthwhile from the students’ perspective.

Furthermore, students particularly enjoyed working with the visual diagnoses and described great associated learning gain and high perceived practical relevance. Successful learning from repeated visual diagnoses has been also described for GPs in the context of tele-dermatology. Through repeated telemedical presentation of conspicuous skin findings to dermatologists, GPs felt more often able to make the correct diagnosis on their own, reducing references to specialists [30,31].

The videos provided in our online clerkship were frequently used and evaluated positively. In some free text comments, even more videos were requested. Students’ preference for videos as e-learning media has been demonstrated earlier, for example, with regard to learning statistics or clinical skills [32,33].

While our ‘additional topics’-material and the possibility for exchange with staff members at university were used by about half of the students, further offers like our ‘gimmicks’ or the possibility for exchanges with fellows were less frequently used. This could be mainly because nearly half of the students in oursample had difficulties completing the tasks in the time allotted. Thus, it seems to be probable that many participants concentrated primarily on the central and mandatory tasks. However, as students’ ratings of some of our optional components were rather negative (‘gimmicks’, exchange with fellows), our results imply that these components are not of central importance for future digital teaching concepts except for enriching variety.

During the synchronous chats with faculty members, we mainly addressed organizational questions, as we assumed students to be unfamiliar with the new format. For future blended learning, this might be unnecessary. Since the personnel effort for synchronous teaching is considerable, it should be carefully assessed whether it is worthwhile. However, if there were to be a stronger focus on discussing professional content related to the practical periods, students could benefit from it. It has been shown that video conferences may enhance the learning success [19].

Despite fellow exchanges having been shown to be a beneficial part of online-teaching concepts [32], our organized discussion forum was hardly used. This might be due to workload as well. Moreover, several students indicated the parallel use of well-established channels for exchange (like messenger services).

As a substitute for the missing work with patients, students were encouraged and sometimes specifically instructed in videos and cases to conduct physical examinations and anamnesis interviews with available persons. Less than a third of the students used this option. This might have been due to time overload of the students during their first completely online ‘Corona-semester’. In a recent survey addressing the change in university teaching in times of COVID-19, 74% of the students confirmed a substantially increased workload [2]. It is also possible that, in addition to the compulsory content, students preferred the more passive and less time-consuming content, such as videos, compared to that which would have required more activity and additional time. Apparently, the overall offer was also too extensive, which is in line with some free text statements.

#### Cohort comparison

Surprisingly, the students’ overall assessment of the digital clerkship was as good as that of the face-to-face clerkship in the two previous semesters. Expectations were equally fulfilled and the effort-benefit ratio was rated comparably good. At the same time, the cohort comparison clearly revealed strengths and weaknesses of the online clerkship. In summary, it can be concluded that structured imparting of theoretical knowledge was perceived as better in the online clerkship, whereas skills and attitudes were reported as being taught better in the face-to-face clerkship. A current review and meta-analysis on online learning in undergraduate medical education showed that online learning has advantages regarding the transfer of knowledge and that there is no indication for less effectiveness [34]. It could even be demonstrated in a pre- and posttest study that students achieve better results through online teaching [35]. Online learning seems to promote student autonomy (self-determined, individual learning in terms of time and place) which might lead to higher motivation and engagement [36]. Furthermore, the ability to design work assignments themselves as well as to design one’s own home as a place of learning were identified as further beneficial factors for online learning [19].

Practical experience through direct patient contact can hardly be adequately replaced online, which has been also reported by another current study on online simulated clinical practice [37]. According to Gormley et al., practical skills can in principle also be learned through online formats, but only in the context of blended learning [33]. It has been shown that digital learning may be just as effective as traditional learning in terms of certain communication skills for medical students [38]. The personal interaction between doctors and patients that can be experienced in a face-to-face clerkship cannot, however, be reproduced virtually in our view, which has been frequently addressed in students’ free text comments and was reported elsewhere [39,40].

The teaching of theoretical content may often be underrepresented in face-to-face GP clerkships, as there is little time in GPs’ everyday practice. In the free text comments on what students liked about the online clerkship, one student stated: ‘*The possibility to really deal intensively with a clinical picture – during everyday practice [there is] often a flood of patients, so that cases often cannot be debriefed in such a structured way*.’

In our study, students completing the online clerkship felt more motivated by their preceptors to become a GP themselves. They also gave better ratings for their preceptors for explaining things during the clerkship. An explanation may be that in our online clerkship GP teachers had to take exclusive ‘quality’ time for communication with the students, whereas in the face-to-face clerkship students sometimes just ‘run along’. This aspect could be considered in conventional clerkships, e.g., by implementing time slots that are dedicated exclusively to student-preceptor communication.

Since both clerkship formats offer clear advantages, a fusion appears to be desirable. It has been stated previously that blended teaching methods represent the future of medical education [40]. The feasibility of blended learning components integrated in a GP clerkship has been already demonstrated [41].

Not only students, but also GP preceptors might benefit from a blended learning as it allows a strongerfocus on supervising practical skills while theoretical background is already covered and can be referred to.

### Strengths and limitations

Online clerkships in undergraduate medical education are an innovative topic of current relevance and insights into Do’s and Don’ts are helpful to guide the development of blended learning concepts, which are expected to be the future of medical education. To our knowledge, we conducted and evaluated the only completely digital GP clerkship during the pandemic in Germany. The use of a wide range of individual online teaching components enabled valuable insights into their different potential to complement future GP clerkships. Response rate for the evaluation of our online clerkship was good for the context of online evaluation and the sample seems to be representative for the German context. Response rates for the evaluation of the two analyzed previous semesters with face-to-face clerkships were very good. The consideration of both quantitative and qualitative data deepened the interpretation of the findings. The opportunity for a cohort comparison with students who completed the clerkship in person strengthens the informative value of our study.

Although good for the context of online evaluation, the response rate in our sample (online semester) must be discussed as a limitation, as participants may be selective. Participation in the paper-pencil evaluations in previous semesters was higher, as students had time to evaluate during the seminar. Higher participation rates for classroom-based evaluations compared to online surveys are known [42]. In addition, it must be considered that students were burdened more than usual by their first ‘Corona semester’ with all its uncertainties and challenges, which may have forced lower participation rates as well. The structurally comparable sample characteristic distributions in our study indicate no systematic sample bias. However, bias by interest or comfort in dealing with the online curriculum cannot be excluded. As a further limitation, it could be discussed that students might have been particularly positive regarding our clerkship. According to students’ direct feedback, we were one out of only a few departments who offered a complex online equivalent on time. This might have led to socially desirable answers or too positive assessments. Furthermore, some of our study results might be limited by the fact that there was no direct comparison between face-to-face and online clerkship by one group of students. Instead, we compared two cohorts having participated either in the face-to-face or the online version only. However, it should be considered that most participants in our online clerkship sample were experienced with face-to-face clerkships as well (70% had completed a 4-week face-to-face GP clerkship previously).

It might also be discussed that the questionnaire used was self-developed and there was no time for pre-testing. However, the questionnaire was developed by a multidisciplinary team with long-standing experience in educational research and a substantial number of questions had been successfully used for many years (face-to-face clerkship). It should also be noted that the assessment of learning gain was based on students’ self-assessments directly after the clerkship. There were no ‘gold standard’ examinations of knowledge and skills to ensure a real comparability of students’ learning gain from online versus face-to-face teaching and to assess long-term effects. Finally, we assume that there is a lot of room for improvement due to the ad hoc development of the complete format (e.g., technical implementation and layout).

### Conclusions and implications for practice and further research

This study adds to literature by showing that undergraduate GP clerkships may benefit from complementing online teaching. Digital components may be used to enhance teaching and increase flexibility during face-to-face clerkships in general practices since they were well adopted and positively evaluated by students. Our results are helpful for developing blended learning concepts by providing information on the potential benefits of different online teaching formats. Especially online clinical case processing, visual diagnoses, and examination videos seem to have the potential to usefully complement GP clerkships. The amount and quality of time during GP clerkships for specific GP teacher-student interactions should be enhanced. Discussions about clinical cases with previous online preparation may be suitable to reach this goal. Practical components of a GP clerkship cannot be replaced by online teaching. In general, it is important to implement new digital content carefully into existing clerkship concepts and to interlock online and face-to-face teaching reasonably (blended learning) instead of simply adding online content ‘on top’. There should be explicit time dedicated to work on online materials to avoid time overload for students. As our results regarding the benefit of individual online teaching formats are based on a completely digital clerkship compared to former face-to-face clerkships, the benefit in the context of blended learning combining both modes needs to be confirmed. Future research should evaluate respective blended learning concepts as well as further teaching formats to examine whether there is added value. The GP teachers’ perspective on combining online and face-to-face teaching should also be explored. Our results are of particular interest for medical faculties, medical teachers, people involved in medical curriculum development, and GPs teaching undergraduates.

## Supplementary Material

Supplemental Material

## Data Availability

Data are available from the authors upon reasonable requests.
