# Supplementary material for: Medical students’ adoption and evaluation of a completely digital general practice clerkship – cross-sectional survey and cohort comparison with face-to-face teaching
Source: Med Educ Online. 2022 Feb 2;27(1):2028334. doi: 10.1080/10872981.2022.2028334 (PMC8812779; doi:10.1080/10872981.2022.2028334)
Supplement: Supplemental Material [file ZMEO_A_2028334_SM6331.docx]

Dear students!

In the following questionnaire we would like to learn more about your experiences and assessments in connection with our online-based mandatory clerkship in general practice. On the one hand, evaluation is particularly important because this teaching format is completely new, and we would like to develop and improve it. On the other hand, we are very interested in comparing the students' assessments with those of the ‘conventional’ mandatory clerkship. Therefore, the survey is a bit more extensive, completing it takes about 10 minutes.

The evaluation is statistically aggregated and will not allow any conclusions about individuals.

Thank you very much for your support!

| **Personal information** | | | | | | | | | | | | | | | | | | | | | | | | | | | | | | | | | |
| --- | --- | --- | --- | --- | --- | --- | --- | --- | --- | --- | --- | --- | --- | --- | --- | --- | --- | --- | --- | --- | --- | --- | --- | --- | --- | --- | --- | --- | --- | --- | --- | --- | --- |
| Age | \|__\|__\| years | Semester (total) | | | \|__\|__\|. semester | | | | | | Sex | | | ○ | | | male | | | | | | ○ | | female | | | | | | | | |
| Have you completed a previous 4-week clerkship in general practice? | | | | | | | | | | | | | | ○ | | | yes | | | | | | ○ | | no | | | | | | | | |
| Is (at least) one of your parents a physician? | | | | | | | | | | | | | | ○ | | | yes | | | | | | ○ | | no | | | | | | | | |
| Do you have family or friends working in general practice? | | | | | | | | | | | | | | ○ | | | yes | | | | | | ○ | | no | | | | | | | | |
| Where did you mainly grow up? | | | | | | ○ | big city | | | | | | | ○ | | | small town | | | | | | ○ | | rural area | | | | | | | | |
| Where can you imagine working in the future?  *(multiple answers are possible)* | | | | | | ○ | big city | | | | | | | ○ | | | small town | | | | | | ○ | | rural area | | | | | | | | |
| I already have a pre-existing completed education in a medical vocational training. | | | | | | | | | | | | | | ○ | | | yes, ___________________________ | | | | | | | | | | | | | | ○ | no | |
| How do you rate yourself in dealing with computers? | | | | | | ○ very fit | | | | | | ○ rather fit | | | | | | | ○ rather unfit | | | | | | | | ○ not fit at all | | | | | | |
| **Information on professional orientation** | | | | | | | | | | | | | | | | | | | | | | | | | | | | | | | | | |
| My current favored career (specialty) is: | | | | | | ○ | Specialist for  General Medicine | | | | | | | ○ | | | Other specialist:  ______________________ | | | | | | | | | ○ | | do not know yet | | | | | |
| For me, to become a specialist in general practice is: | | | | | | ○ | the *favored* career option | | | | | | | ○ | | | an *imaginable* career option | | | | | | | | | ○ | | *no* career option | | | | | |
| Working in ambulant health care for me is: | | | | | | ○ | the *favored* career option | | | | | | | ○ | | | an *imaginable* career option | | | | | | | | | ○ | | *no* career option | | | | | |
| Working as a self-employed physician in my own private practice later on is: | | | | | | ○ | the *favored* career option | | | | | | | ○ | | | an *imaginable* career option | | | | | | | | | ○ | | *no* career option | | | | | |
| **Overall assessment of the online-based mandatory clerkship in general medicine** | | | | | | | | | | | | | | | | | | | | | | | | | | | | | | | | | |
|  | | | | | | | | | | | | | *I completely agree* | | | | | *I rather agree* | | | | | | *I rather disagree* | | | | | | *I completely disagree* | | | |
| I enjoyed the online-based mandatory clerkship. | | | | | | | | | | | | | ○ | | | | | ○ | | | | | | ○ | | | | | | ○ | | | |
| The online-based mandatory clerkship was well structured. | | | | | | | | | | | | | ○ | | | | | ○ | | | | | | ○ | | | | | | ○ | | | |
| The processing of the online-based mandatory clerkship was intuitive. | | | | | | | | | | | | | ○ | | | | | ○ | | | | | | ○ | | | | | | ○ | | | |
| The online-based clerkship was easy to work on in the time allotted. | | | | | | | | | | | | | ○ | | | | | ○ | | | | | | ○ | | | | | | ○ | | | |
| The online-based mandatory clerkship was multifaceted. | | | | | | | | | | | | | ○ | | | | | ○ | | | | | | ○ | | | | | | ○ | | | |
| The online-based mandatory clerkship had a high practical relevance. | | | | | | | | | | | | | ○ | | | | | ○ | | | | | | ○ | | | | | | ○ | | | |
| I have learned a lot through the online-based clerkship. | | | | | | | | | | | | | ○ | | | | | ○ | | | | | | ○ | | | | | | ○ | | | |
| The online-based mandatory clerkship provided good insights into general practice activities. | | | | | | | | | | | | | ○ | | | | | ○ | | | | | | ○ | | | | | | ○ | | | |
| The online-based mandatory clerkship was able to convey the special features of general practice activities. | | | | | | | | | | | | | ○ | | | | | ○ | | | | | | ○ | | | | | | ○ | | | |
| The online-based mandatory clerkship has increased my interest in general practice. | | | | | | | | | | | | | ○ | | | | | ○ | | | | | | ○ | | | | | | ○ | | | |
| The tasks in the online-based mandatory clerkship were set in an understandable way. | | | | | | | | | | | | | ○ | | | | | ○ | | | | | | ○ | | | | | | ○ | | | |
| I liked the possibility of having relatively free time to work on the mandatory clerkship. | | | | | | | | | | | | | ○ | | | | | ○ | | | | | | ○ | | | | | | ○ | | | |
| The online-based mandatory clerkship should also complement medical studies in times of ‘normal teaching’. | | | | | | | | | | | | | ○ | | | | | ○ | | | | | | ○ | | | | | | ○ | | | |
| **Usage behavior** | | | | | | | | | | | | | | | | | | | | | | | | | | | | | | | | |  |
| Which of the following individual components of the online-based mandatory clerkship in general practice have you used?  *(multiple answers are possible)* | | | ○ | Clinical (SOAP-)cases | | | | ○ | Info videos (e.g., introductory video, interview with a medical assistant) | | | | | | | | | | | ○ | Exchange with fellow students in the forum of the ‘student portal’ | | | | | | | | | | | |  |
|  |  |  | ○ | Visual diagnoses | | | | ○ | Chat with members of the department of general practice | | | | | | | | | | | ○ | Additional topics (e.g., compliance, living will) | | | | | | | | | | | |  |
|  |  |  | ○ | Examination videos (e.g., vertigo, check-up) | | | | ○ | Communication with the GP teacher (e.g., e-mail, telephone) | | | | | | | | | | | ○ | Gimmicks (e.g., puzzles) | | | | | | | | | | | |  |
| What electronic device did you mainly use for the online-based mandatory clerkship? *(multiple answers are possible)* | | | | | | | | | | ○ Laptop | | | | | | ○ Desktop PC | | | | | | ○ Tablet computer | | | | | | | ○ Smartphone | | | | |
| Which communication channels did you mainly use to communicate with your GP teacher? *(multiple answers are possible)* | | | | | | | | | | ○ Video consultation hour | | | | | | ○ Other video chat | | | | | | ○ Telephone | | | | | | | ○ E-mail | | | | |
| **Please comment on the following statements:** | | | | | | | | | | *yes* | | | | | | *rather yes* | | | | | | *rather no* | | | | | | | *no* | | | | |
| I worked on the contents of the online-based mandatory clerkship daily as intended. | | | | | | | | | | ○ | | | | | | ○ | | | | | | ○ | | | | | | | ○ | | | | |
| I worked on the contents of the online-based mandatory clerkship chronologically. | | | | | | | | | | ○ | | | | | | ○ | | | | | | ○ | | | | | | | ○ | | | | |
| I worked on the online-based mandatory clerkship together with other students. | | | | | | | | | | ○ | | | | | | ○ | | | | | | ○ | | | | | | | ○ | | | | |
| I conducted physical examinations on others (e.g., partner, flatmate). | | | | | | | | | | ○ | | | | | | ○ | | | | | | ○ | | | | | | | ○ | | | | |
| I had an exchange with learning partners about the contents of the online-based mandatory clerkship. | | | | | | | | | | ○ | | | | | | ○ | | | | | | ○ | | | | | | | ○ | | | | |
| **Assessment of the individual components of the online-based mandatory clerkship** | | | | | | | | | | | | | | | | | | | | | | | | | | | | | | | | | |
| **A – Working enjoyment** | | | | | | | | | | | | | | | | | | | | | | | | | | | | | | | | | |
| **I enjoyed working on the component ...** | | | | | | | | | | *I completely agree* | | | | | *I rather*  *agree* | | | | | | *I rather disagree* | | | | | | *I completely disagree* | | | | | | |
| … Clinical (SOAP-)cases | | | | | | | | | | ○ | | | | | ○ | | | | | | ○ | | | | | | ○ | | | | | | |
| … Visual diagnoses | | | | | | | | | | ○ | | | | | ○ | | | | | | ○ | | | | | | ○ | | | | | | |
| … Examination videos (e.g., vertigo, check-up) | | | | | | | | | | ○ | | | | | ○ | | | | | | ○ | | | | | | ○ | | | | | | |
| … Info videos (e.g., introductory video, interview with a medical assistant) | | | | | | | | | | ○ | | | | | ○ | | | | | | ○ | | | | | | ○ | | | | | | |
| … Chat with members of the department of general practice | | | | | | | | | | ○ | | | | | ○ | | | | | | ○ | | | | | | ○ | | | | | | |
| … Communication with the GP teacher (e.g., e-mail, telephone) | | | | | | | | | | ○ | | | | | ○ | | | | | | ○ | | | | | | ○ | | | | | | |
| ... Exchange with fellow students in the forum of the ‘student portal’ | | | | | | | | | | ○ | | | | | ○ | | | | | | ○ | | | | | | ○ | | | | | | |
| … Additional topics (e.g., compliance, living will) | | | | | | | | | | ○ | | | | | ○ | | | | | | ○ | | | | | | ○ | | | | | | |
| … Gimmicks (e.g., puzzles) | | | | | | | | | | ○ | | | | | ○ | | | | | | ○ | | | | | | ○ | | | | | | |
| **B – Learning gain** | | | | | | | | | | | | | | | | | | | | | | | | | | | | | | | | | |
| **Through the component I had a high learning gain ...** | | | | | | | | | | *I completely agree* | | | | | *I rather*  *agree* | | | | | | *I rather disagree* | | | | | | *I completely disagree* | | | | | | |
| … Clinical (SOAP-)cases | | | | | | | | | | ○ | | | | | ○ | | | | | | ○ | | | | | | ○ | | | | | | |
| … Visual diagnoses | | | | | | | | | | ○ | | | | | ○ | | | | | | ○ | | | | | | ○ | | | | | | |
| … Examination videos (e.g., vertigo, check-up) | | | | | | | | | | ○ | | | | | ○ | | | | | | ○ | | | | | | ○ | | | | | | |
| … Info videos (e.g., introductory video, interview with a medical assistant) | | | | | | | | | | ○ | | | | | ○ | | | | | | ○ | | | | | | ○ | | | | | | |
| … Chat with members of the department of general practice | | | | | | | | | | ○ | | | | | ○ | | | | | | ○ | | | | | | ○ | | | | | | |
| … Communication with the GP teacher (e.g., e-mail, telephone) | | | | | | | | | | ○ | | | | | ○ | | | | | | ○ | | | | | | ○ | | | | | | |
| ... Exchange with fellow students in the forum of the ‘student portal’ | | | | | | | | | | ○ | | | | | ○ | | | | | | ○ | | | | | | ○ | | | | | | |
| … Additional topics (e.g., compliance, living will) | | | | | | | | | | ○ | | | | | ○ | | | | | | ○ | | | | | | ○ | | | | | | |
| … Gimmicks (e.g., puzzles) | | | | | | | | | | ○ | | | | | ○ | | | | | | ○ | | | | | | ○ | | | | | | |
| **C – Practical relevance** | | | | | | | | | | | | | | | | | | | | | | | | | | | | | | | | | |
| **The component had a high practical relevance ...** | | | | | | | | | | *I completely agree* | | | | | *I rather*  *agree* | | | | | | *I rather*  *disagree* | | | | | | *I completely disagree* | | | | | | |
| … Clinical (SOAP-)cases | | | | | | | | | | ○ | | | | | ○ | | | | | | ○ | | | | | | ○ | | | | | | |
| … Visual diagnoses | | | | | | | | | | ○ | | | | | ○ | | | | | | ○ | | | | | | ○ | | | | | | |
| … Examination videos (e.g., vertigo, check-up) | | | | | | | | | | ○ | | | | | ○ | | | | | | ○ | | | | | | ○ | | | | | | |
| … Info videos (e.g., introductory video, interview with a medical assistant) | | | | | | | | | | ○ | | | | | ○ | | | | | | ○ | | | | | | ○ | | | | | | |
| … Chat with members of the department of general practice | | | | | | | | | | ○ | | | | | ○ | | | | | | ○ | | | | | | ○ | | | | | | |
| … Communication with the GP teacher (e.g., e-mail, telephone) | | | | | | | | | | ○ | | | | | ○ | | | | | | ○ | | | | | | ○ | | | | | | |
| ... Exchange with fellow students in the forum of the ‘student portal’ | | | | | | | | | | ○ | | | | | ○ | | | | | | ○ | | | | | | ○ | | | | | | |
| … Additional topics (e.g., compliance, living will) | | | | | | | | | | ○ | | | | | ○ | | | | | | ○ | | | | | | ○ | | | | | | |
| … Gimmicks (e.g., puzzles) | | | | | | | | | | ○ | | | | | ○ | | | | | | ○ | | | | | | ○ | | | | | | |
| **D – Insight into the work of a general practitioner** | | | | | | | | | | | | | | | | | | | | | | | | | | | | | | | | | |
| **The component gave me an insight into the work of a general practitioner ...** | | | | | | | | | | *I completely agree* | | | | | *I rather*  *agree* | | | | | | *I rather*  *disagree* | | | | | | *I completely disagree* | | | | | | |
| … Clinical (SOAP-)cases | | | | | | | | | | ○ | | | | | ○ | | | | | | ○ | | | | | | ○ | | | | | | |
| … Visual diagnoses | | | | | | | | | | ○ | | | | | ○ | | | | | | ○ | | | | | | ○ | | | | | | |
| … Examination videos (e.g., vertigo, check-up) | | | | | | | | | | ○ | | | | | ○ | | | | | | ○ | | | | | | ○ | | | | | | |
| … Info videos (e.g., introductory video, interview with a medical assistant) | | | | | | | | | | ○ | | | | | ○ | | | | | | ○ | | | | | | ○ | | | | | | |
| … Chat with members of the department of general practice | | | | | | | | | | ○ | | | | | ○ | | | | | | ○ | | | | | | ○ | | | | | | |
| … Communication with the GP teacher (e.g., e-mail, telephone) | | | | | | | | | | ○ | | | | | ○ | | | | | | ○ | | | | | | ○ | | | | | | |
| ... Exchange with fellow students in the forum of the ‘student portal’ | | | | | | | | | | ○ | | | | | ○ | | | | | | ○ | | | | | | ○ | | | | | | |
| … Additional topics (e.g., compliance, living will) | | | | | | | | | | ○ | | | | | ○ | | | | | | ○ | | | | | | ○ | | | | | | |
| … Gimmicks (e.g., puzzles) | | | | | | | | | | ○ | | | | | ○ | | | | | | ○ | | | | | | ○ | | | | | | |

Attention: The following questions serve as a comparison with the evaluations of previous years of mandatory clerkships. Please answer all questions completely, even if they seem to be duplications. **Please also note the changed answer format.**

| **General** | totally |  | not at all |
| --- | --- | --- | --- |
| My expectations regarding the goals and topics of the mandatory clerkship have been fulfilled. | ① ② ③ ④ ⑤ ⑥ | | |
| During the mandatory clerkship I learned professionally. | ① ② ③ ④ ⑤ ⑥ | | |
| During the mandatory clerkship, there was the opportunity for a professional exchange with my GP teacher. | ① ② ③ ④ ⑤ ⑥ | | |
| The mandatory clerkship encouraged me to further deepen my self-study of the topics covered. | ① ② ③ ④ ⑤ ⑥ | | |
| Measured in terms of time and organizational effort, participation in the mandatory clerkship was worthwhile. | ① ② ③ ④ ⑤ ⑥ | | |
| **How well or poorly were you taught new skills or attitudes in your mandatory clerkship on the following topics?** | very well | | very poor |
| Detection of common diseases in general practice and their therapy | ① ② ③ ④ ⑤ ⑥ | | |
| Prescriptions (prescriptions, medicines, physiotherapy, incapacity to work, etc.) | ① ② ③ ④ ⑤ ⑥ | | |
| Preventive measures (prevention of a later serious illness, e.g., hypercholesterolemia, obesity) | ① ② ③ ④ ⑤ ⑥ | | |
| Screening measures (preventive examinations, e.g., check-ups, cancer screening) | ① ② ③ ④ ⑤ ⑥ | | |
| Importance of family medicine | ① ② ③ ④ ⑤ ⑥ | | |
| Home visits (indication, procedure, frequency) | ① ② ③ ④ ⑤ ⑥ | | |
| Communication/conversation skills (also with difficult patients, compliance problems) | ① ② ③ ④ ⑤ ⑥ | | |
| Meeting patient expectations | ① ② ③ ④ ⑤ ⑥ | | |
| Care of the chronically ill | ① ② ③ ④ ⑤ ⑥ | | |
| Vaccinations | ① ② ③ ④ ⑤ ⑥ | | |
| Physical examination techniques | ① ② ③ ④ ⑤ ⑥ | | |
| Instrumental diagnostics in general practice | ① ② ③ ④ ⑤ ⑥ | | |

| **Questions about contact with the supervising GP teacher** | totally | not at all |
| --- | --- | --- |
| I was motivated by the teacher to become a general practitioner myself later. | ① ② ③ ④ ⑤ ⑥ ➆ ➇ ➈ ➉ | |
| The GP teacher was able to explain everything well to me. | ① ② ③ ④ ⑤ ⑥ ➆ ➇ ➈ ➉ | |
| **At the end** | totally | not at all |
| The clerkship was far too theoretical. | ① ② ③ ④ ⑤ ⑥ ➆ ➇ ➈ ➉ | |
| The demands on me were too high. | ① ② ③ ④ ⑤ ⑥ ➆ ➇ ➈ ➉ | |
| Overall, I am satisfied with the quality of the mandatory clerkship. | ① ② ③ ④ ⑤ ⑥ ➆ ➇ ➈ ➉ | |

| What did you like about the online-based mandatory clerkship in general practice? |
| --- |
| In your opinion, how could the online-based mandatory clerkship in general practice be further improved? |
| Would you like to add something? |

**Thanks for your support!**
